# Genomic and Transcriptional Co-Localization of Protein-Coding and Long Non-Coding RNA Pairs in the Developing Brain

**DOI:** 10.1371/journal.pgen.1000617

**Published:** 2009-08-21

**Authors:** Jasmina Ponjavic, Peter L. Oliver, Gerton Lunter, Chris P. Ponting

**Affiliations:** MRC Functional Genomics Unit, Department of Physiology, Anatomy and Genetics, University of Oxford, Oxford, United Kingdom; RIKEN Genomic Sciences Center, Japan

## Abstract

Besides protein-coding mRNAs, eukaryotic transcriptomes include many long non-protein-coding RNAs (ncRNAs) of unknown function that are transcribed away from protein-coding loci. Here, we have identified 659 intergenic long ncRNAs whose genomic sequences individually exhibit evolutionary constraint, a hallmark of functionality. Of this set, those expressed in the brain are more frequently conserved and are significantly enriched with predicted RNA secondary structures. Furthermore, brain-expressed long ncRNAs are preferentially located adjacent to protein-coding genes that are (1) also expressed in the brain and (2) involved in transcriptional regulation or in nervous system development. This led us to the hypothesis that spatiotemporal co-expression of ncRNAs and nearby protein-coding genes represents a general phenomenon, a prediction that was confirmed subsequently by *in situ* hybridisation in developing and adult mouse brain. We provide the full set of constrained long ncRNAs as an important experimental resource and present, for the first time, substantive and predictive criteria for prioritising long ncRNA and mRNA transcript pairs when investigating their biological functions and contributions to development and disease.

## Author Summary

Virtually all of the eukaryotic genome is transcribed, yet far from all transcripts encode protein. Very little is known about the functions of most non-coding transcripts or, indeed, whether they convey functions at all. Among all such transcripts, we have chosen to consider long non-coding RNAs (ncRNAs) that are transcribed outside of known protein-coding gene loci. Our approach has focused on mouse long ncRNAs whose genomic sequences are conserved in humans, and also on ncRNAs that are expressed in the brain. This conservation might reflect the functionality of the underlying DNA, rather than the ncRNA, sequence. However, this cannot fully explain the concentration of predicted RNA structures in these ncRNAs. These long ncRNAs also tend to be transcribed in the genomic neighbourhood of protein-coding genes whose functions relate to transcription or to nervous system development. These observations are consistent with the positive transcriptional regulation in *cis* of these genes with nearby transcription of ncRNAs. This model implies co-expression of protein-coding and noncoding transcripts, a hypothesis that we validated experimentally. These findings are particularly important because they provide a rationale for prioritising specific ncRNAs when experimentally investigating regulation of protein-coding gene expression.

## Introduction

The mammalian genome displays a complex and extensive pattern of interlaced transcription of protein-coding genes and thousands of non-coding RNA (ncRNA; see Materials and Methods for definitions) loci [1]. Exons from ncRNA loci may overlap on the same (*sense*), or opposite (*antisense*), strand with exons from other transcripts, including those from protein-coding genes. They may also be contained within introns of other transcripts. Other ncRNAs are transcribed from *bidirectional* promoters: their transcriptional events, and those for neighbouring transcripts from the opposite strand, are initiated in close genomic proximity. Several recent studies investigated whether *cis*-antisense, intronic, or bidirectional ncRNAs regulate the transcription of protein-coding genes whose loci they overlap [2],[3]. These report complex relationships between the expression profiles of ncRNAs and their overlapping protein-coding genes in adult mice. Further investigations, however, are clearly needed to investigate other types of ncRNAs, in particular *intergenic* and long (>200 nt) ncRNAs transcribed from outside protein-coding loci, and those expressed during development.

If most long ncRNAs convey biological functions, then what these molecular mechanisms are remain almost completely unknown. For the few with established mechanisms a general theme has emerged of them acting as transcriptional regulators of protein-coding genes (reviewed in [4]). For many such ncRNAs, the genomic location of their transcription has proved key to their mechanism. When promoters of non-coding and coding transcripts are closely juxtaposed on the chromosome, for example, then transcriptional events initiated from them may be coupled. This has been shown to occur following chromatin remodelling of chromosomal domains [5]–[7], or because of collisions between transcriptional machineries processing along sequence in close proximity [8], or because of transcriptional interference when transcription proceeds through a promoter sequence thereby suppressing transcription initiation from it [8]. Other long ncRNAs are *cis*-regulators of transcription via indirect means involving their participation in ribonucleoprotein complexes [9],[10]. Other long ncRNAs, such as NRON or 7SK, act in *trans*: they regulate the expression of target genes or gene products from chromosomes other than the ones from which they are transcribed [11]–[13].

*Cis*-regulation by ncRNAs of protein-coding gene transcription is well-established in imprinting [14] and for developmental genes, such as *Dlx5* and *Dlx6* [9], yet these represent transcriptional events that overlap on the genome. By way of contrast, we sought statistical evidence that pairs of adjacent, yet distinct, coding and non-coding loci often give rise to separate transcripts with similar spatiotemporal expression patterns indicative of positive co-operativity of transcriptional regulation. (Of course, negative co-operativity by, for example, transcriptional interference is also likely. However, such instances tend to be harder to establish experimentally owing to low levels of ncRNA expression.) We considered that if evidence of transcriptional co-operativity were to be forthcoming then specific pairs of coding and noncoding transcripts could be prioritised for experimentation. In such studies, it is important to demonstrate that long ncRNAs and mRNAs are transcribed exclusively from separate promoters. Otherwise, similarities in their expression profiles may not represent distinct transcriptional events but instead single transcripts spanning both coding and noncoding exons.

We recently demonstrated several evolutionary signatures of functionality for a large set of mouse long ncRNAs and their promoters [15]. These long ncRNA sequences are largely full-length [16], map to genomic loci lying outside of protein-coding gene models and consequently are unlikely to act as antisense transcripts of a neighbouring gene locus. Although some of these ncRNAs may result from uncoordinated and inconsequential transcription, evidence of transcriptional regulation [17] and constraints on splicing motifs [15] cannot be explained by such transcriptional ‘noise’.

We were interested in whether long intergenic ncRNAs are located randomly with respect to protein-coding genes. If not, this might suggest a trend for long ncRNAs to act in *cis* with neighbouring protein-coding genes. To improve our chances of detecting non-uniformities of chromosomal location, we considered long ncRNAs whose genomic sequences are evolutionarily constrained and thus are more likely to be functional. If long ncRNAs possess, in general, *cis*-regulatory roles, one might expect their transcribed genomic regions to lie in proximity to their functionally-linked protein-coding genes, and their tissue expression profiles to be similar. Finally, it might also be expected that functional long ncRNAs would tend to be linked to certain subsets of protein-coding genes that convey particular biological functions.

We investigated this *cis*-regulatory hypothesis for a set of 659 evolutionary constrained long ncRNAs and found large-scale and experimental evidence for co-regulation of non-coding and protein-coding transcript pairs. For the first time, we show that these constrained long ncRNAs are not evenly distributed on the genome but rather tend to be concentrated near to genes with similar expression patterns and from particular functional classes. These findings immediately provide new and unbiased criteria for prioritising long ncRNAs for experimental investigation. Hundreds of constrained long ncRNAs can now be targeted for detailed examination, specifically those that either (*i*) are expressed in the brain during development and are transcribed in proximity to transcription factor genes, or (*ii*) are expressed outside of the CNS in adult individuals and that lie adjacent to signalling genes.

## Results

This study examined large numbers of mouse long intergenic ncRNAs, partitioned by the availability or otherwise of evidence for their expression in the brain or during development, and of evidence for sequence constraint. Previous studies had focused specifically on the expression of antisense, bidirectional and intronic ncRNAs in 56 day old adult mice or during mouse embryonic stem cell differentiation [2],[3]. For each set of ncRNA loci we examined the null hypothesis that they are located at random relative to protein-coding genes. Instead, we find strong and significant co-expression and functional biases. We show experimentally that these biases do not derive from single transcriptional events.

### Constrained ncRNAs are enriched in predicted RNA secondary structures

**Figure 1:**
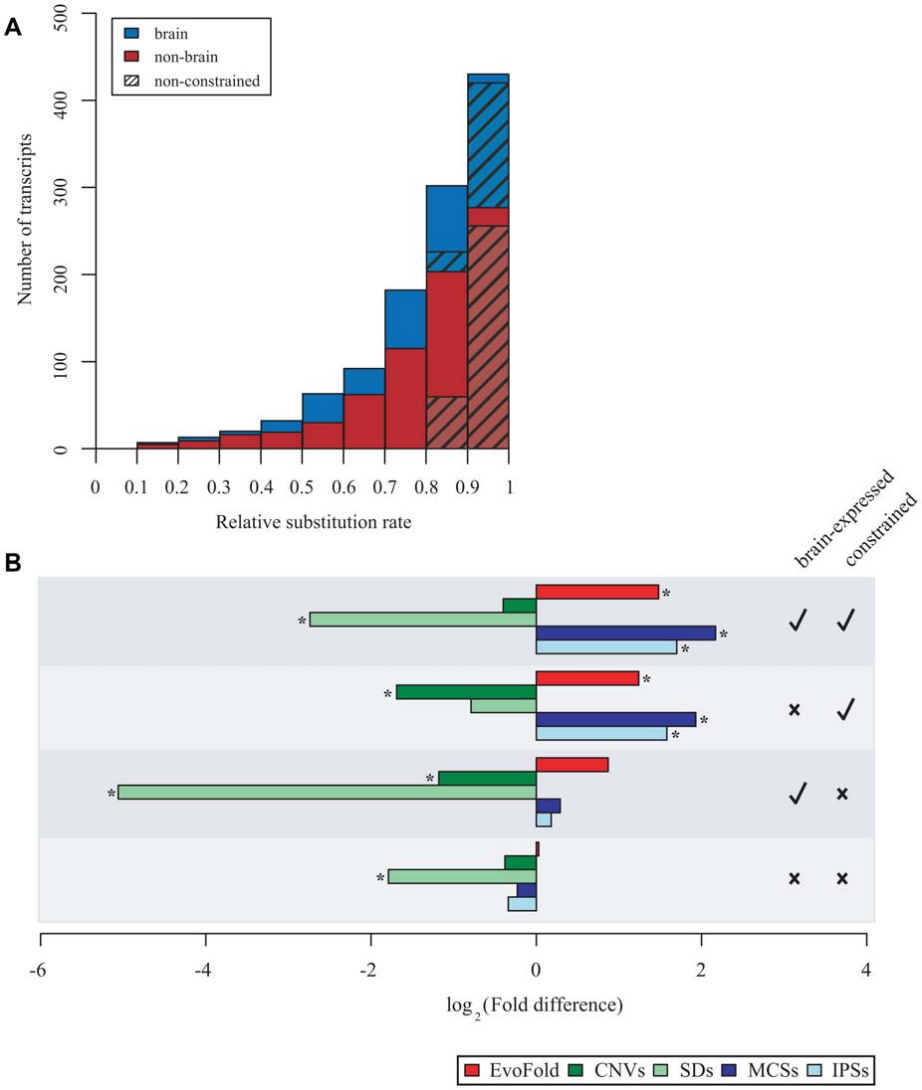
A set of 659 non-coding RNA (ncRNA) transcripts, where each exhibits evidence of constraint on nucleotide substitutions since the mouse-human last common ancestor, shows significant enrichments in sequence predicted to contain folded RNA structures. (A) An aggregated histogram showing 1,113 ncRNAs whose relative substitution rates (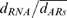) in mouse-human comparisons could be estimated reliably (see Materials and Methods). Each bin provides the number of ncRNAs whose relative substitution rate falls within a given (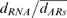) interval. Brain-expressed ncRNAs are indicated in blue, non-brain-expressed ncRNAs in red, and ncRNAs that exhibit significantly reduced substitution rates are represented as non-shaded bars. Of all ncRNAs with relative substitution rates between 0.9 and 1.0, 93% exhibit rates that are not significantly different from likely selectively neutral sequence and were, therefore, classified as non-constrained (shaded bars). (B) Evofold-predicted RNA secondary structures (red bars) and conserved sequence (of two types: either PhastCons multispecies conserved elements [MCSs; dark blue] or indel-purified segments [IPSs; light blue]) are each significantly enriched within constrained long ncRNAs. Such ncRNAs also tend to be depleted within segmentally duplicated (SDs; light green) and human copy number variable (CNVs; dark green) sequence. Checkmarks and crosses indicate whether there is evidence for long ncRNAs to be expressed in the brain and to show sequence constraint (see main text). The fold difference (X-axis) is shown on a log_2_-scale. An asterisk (*) indicates that a ncRNA set is significantly enriched/depleted in an annotation when compared with annotation densities in G+C-matched and randomly-sampled sequences (*p*<2×10^−4^).

We started by analysing 3,122 long ncRNAs transcribed from intergenic regions (see Materials and Methods) that, when considered together, exhibit evolutionary constraint [15]. Among these ncRNAs, we then identified 659 long ncRNAs that individually show evidence of constraint (hereafter termed *constrained* long ncRNAs): individually, their mouse-human nucleotide substitution rate is significantly (*p*<2.5×10^−2^) suppressed relative to rates for neighbouring transposable elements (Figure 1A; see Materials and Methods). As expected from these suppressed rates, many of these constrained long ncRNA loci (for example, AK034244, AK034417, AK039739, and AK048867) are alignable to the genomes of more distantly-related species, such as chicken. Henceforth, we focus on these 659 constrained ncRNAs since they are more likely to be functional, and less likely to represent random transcriptional events. Indeed, this is consistent with constrained ncRNAs being more frequently supported by CAGE (Cap-analysis gene expression) tag evidence [1],[18] than are non-constrained ncRNAs (319/659, 48% *versus* 537/1932, 28%, respectively; *p*<10^−4^, χ^2^-test).

Suppression of nucleotide substitution rates for these 659 ncRNAs would be compatible with functional roles for their underlying genomic DNA sequences, rather than their transcripts, for example if their transcription elongation remodels chromatin structure thereby causing conserved DNA sequence motifs to become more accessible to transcription factors. Evidence that the RNA transcript itself is often functional comes from the significant 2.4- to 2.8-fold over-representation of predicted stable RNA secondary structures within constrained ncRNAs (*p*<10^−4^) (Figure 1B); 178 of 659 constrained long ncRNAs contain at least one predicted RNA secondary structure. A previous study [2] also proposed that a large proportion (39%) of brain-expressed ncRNAs contain predicted RNA secondary structures. Figure S1 illustrates three such likely functional ncRNA molecules (AK082637, AK082142 and AK032637), each expressed in the developing mouse brain, which contain predicted RNA secondary structures.

In summary, ncRNA sequences that have most frequently experienced purifying selection of substitution, duplication and insertion or deletion mutations (Figure 1B) tend to possess a higher than expected proportion of predicted folded RNA structures.

### Constrained ncRNAs expressed during mouse development cluster close to transcriptional regulator genes

Next, we investigated whether long ncRNA loci tend to be transcribed adjacent to protein-coding genes associated with particular sets of molecular functions. If so, we reasoned that such pairings might reflect neighbouring non-protein-coding and protein-coding transcripts that act by regulating each other's transcription. For this study, long ncRNAs derived from mouse brain (see Materials and Methods) were considered separately from other long ncRNAs since their genomic sequences are more frequently conserved, and thus more likely to show conserved functions (Table S1). More specifically, brain-expressed long ncRNAs exhibit a significantly greater proportion of bases aligned to orthologous human sequence than long ncRNAs derived from other tissues (*p* = 2×10^−4^; Kolmogorov-Smirnov two-sided test).

**Figure 2:**
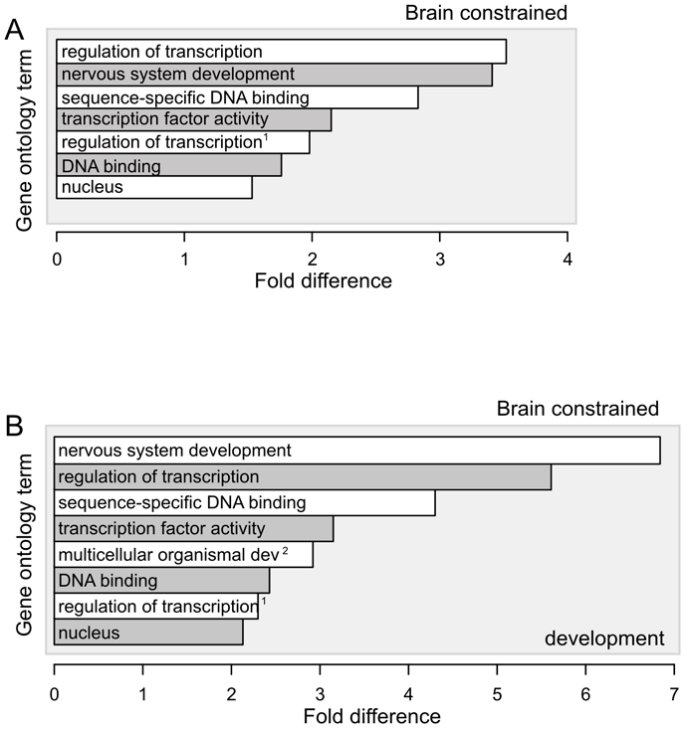
Brain-derived ncRNAs, in particular those expressed during development, tend to lie adjacent to protein-coding genes that are involved in transcriptional regulation during development. (A) Shown are fold-enrichments (X-axis) of Gene Ontology (GO) terms (Y-axis) for constrained brain-expressed ncRNAs. (B) Brain-derived ncRNAs that are expressed during mouse embryonic or neonatal *development* show significant tendencies to be proximal to transcription factor and developmental protein-coding genes, whereas those expressed in adult mice show no significant associations (not shown). (A, B) GO terms are listed if they are over-represented among protein-coding genes proximal to ncRNAs compared to those proximal to randomly-sampled sequences (*p*<10^−3^, EFDR = 0.08 entries). The fold difference (X-axis) is calculated between observed densities of ncRNAs associated with GO terms of nearby protein-coding genes and expected densities of corresponding G+C-matched and randomly sampled sequences. Abbreviations: ^1^ regulation of transcription, DNA dependent, ^2^ multicellular organismal development.

In support of our *cis*-regulation hypothesis, we find that the 239 brain-expressed ncRNA loci are not evenly distributed along the mouse genome. Instead, they exhibit significant preferences (∼2 to 3-fold enrichments) to be closest to protein-coding genes from two functional classes, namely genes that are involved either in transcriptional regulation or in nervous system development (Figure 2A). Importantly, these functional associations were significant only for the set of long ncRNAs that are expressed in the developing mouse brain (∼2 to 7-fold enrichments; Figure 2B), and thus were absent for the set of long ncRNAs expressed in the adult brain. For these studies, results are highly significant (*p*<10^−3^) and a low number of chance associations is expected (estimated number of false discovery observations = 0.08 annotations). These statistical tests took care to account for variations arising from known chromosome-specific and G+C biases (see Materials and Methods).

**Figure 3:**
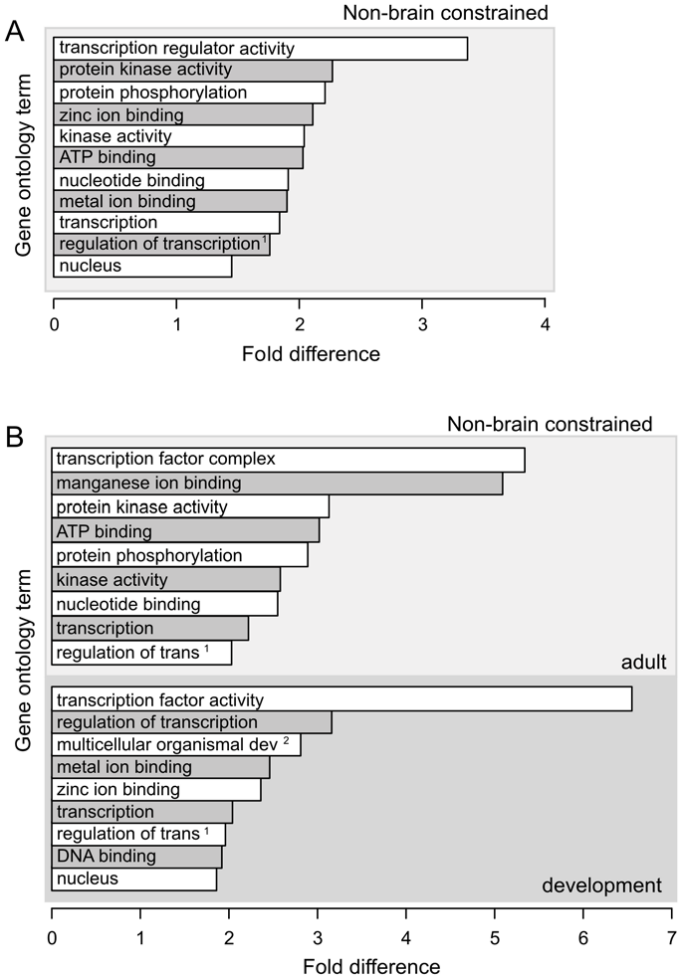
Non-brain-derived ncRNAs, in particular those expressed in adult mice, tend to be transcribed adjacent to protein-coding genes involved in signal transduction pathways. (A) Shown are fold-enrichments (X-axis) of Gene Ontology (GO) terms (Y-axis) for non-brain-expressed ncRNAs that are evolutionarily constrained. (B) Non-brain-derived ncRNAs that are either expressed in *adult* mice (upper subpanel, light gray) or during mouse embryonal or neonatal *development* (lower subpanel, dark gray) show significant tendencies to be proximal to protein-coding genes with protein kinase, transcription factor and developmental GO annotations. (A, B) GO terms are listed if they are over-represented among protein-coding genes proximal to ncRNAs compared to those proximal to randomly-sampled sequences (*p*<10^−3^, EFDR = 0.08 entries). The fold difference (X-axis) is calculated between observed densities of ncRNAs associated with GO terms of nearby protein-coding genes and expected densities of corresponding G+C-matched and randomly sampled sequences. Abbreviations: ^1^ regulation of transcription, DNA dependent, ^2^ multicellular organismal development. Kinase and phosphatase genes strongly contribute to the observed enrichments seen for metal ion-, or ATP-, or manganese ion-binding.

Long ncRNAs expressed outside of the brain, on the other hand, exhibit a strong and significant (∼2-fold increase; *p*<10^−3^) tendency to be transcribed adjacent to protein-coding genes involved in protein kinase-mediated signalling pathways (Figure 3A). This particular preference is apparent for transcripts expressed only in adult, but not in the developing, brain. Finally, the bias for long ncRNA loci to be transcribed adjacent to genes encoding transcription regulators holds true for transcripts that are expressed in developing non-brain, as well as brain, tissues (Figure 3B).

Next, by comparing promoter sequences of these long ncRNA loci, predicted using CAGE clusters [18], to those of neighbouring protein-coding genes, we found evidence that the ncRNAs tend to be expressed in limited tissue repertoires, whereas their partner protein-coding genes tend to be expressed more widely. Only a third of constrained long ncRNAs have CpG-associated promoters (107 of 319), compared with 72% of all protein-coding genes [19], and thus most are expected to be expressed in a limited repertoire of tissues. By contrast, promoters of protein-coding genes that neighbour long ncRNA loci are depleted in TATA-promoters (data not shown), instead belonging predominantly to the Broad class [18] which are often associated with CpG islands and with housekeeping or brain-specific genes [20]. Furthermore, the initiator (Inr) element or Cap motif [18] of these neighbouring protein-coding genes is composed mainly of PyPu dinucleotides (CA, CG and TG; Figure S2) which are known to be associated with high-expression levels, whereas for the long ncRNAs it is mainly PuPu (GA and GG; Figure S2), which is favoured in rarely-expressed transcripts [18].

Finally, we investigated whether the tissue specificity of protein-coding genes differed according to whether their genomically adjacent long ncRNA loci are evolutionarily constrained or are expressed in the brain. For this we took advantage of a relative entropy (RE; Kullback-Leibler distance) measure based on the distribution of CAGE tags from different tissues [21]. We found that protein-coding genes located adjacent to brain-expressed and constrained long ncRNA loci exhibit significantly higher tissue specificity (median RE = 0.63) than coding genes either adjacent to unconstrained long ncRNA loci (median RE = 0.45) or adjacent to constrained long ncRNA loci expressed in non-brain tissues (median RE = 0.52) (Kolmogorov-Smirnov test, *p*≤0.05).

These results are thus consistent with transcription of constrained ncRNAs during brain development often regulating transcription of genomically adjacent protein-coding transcription factor genes in a tissue-specific manner.

### Tissue co-expression and directional transcriptional preference of non-coding and protein-coding transcript pairs

**Figure 4:**
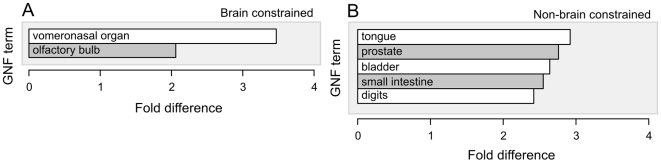
Brain-derived ncRNAs tend to transcribed adjacent to protein-coding genes with high expression in the mouse vomeronasal organ and olfactory bulb. Shown are brain- (A) and non-brain-expressed (B) ncRNAs that are evolutionarily constrained. The Y-axis represents tissues in which protein-coding genes located in proximity to a ncRNA are expressed at unusually high levels [57] (see Materials and Methods). ncRNAs are significantly associated with protein-coding genes that are expressed in these tissues (Y-axis) when compared to randomly sampled G+C matched sequence (*p*<10^−3^, EFDR = 0.05 entries). The significant fold increase is shown on the X-axis. Non-brain-derived ncRNAs tend to be in close proximity to protein-coding genes expressed in tongue, prostate, intestine and digits, while brain-expressed ncRNAs tend to be located near protein-coding genes expressed in the vomeronasal organ and olfactory bulb. Similar results are found when ncRNAs are partitioned by their expression in brain or in non-brain tissues during development (Table S2).

A prediction of this model is that neighbouring protein-coding and ncRNA transcripts are more likely to be expressed in the same tissue than by chance alone. Upon testing this prediction we found that brain-expressed long ncRNA loci did indeed show a 2 to 3-fold significant tendency to neighbour protein-coding genes that are highly expressed in brain-associated tissues, particularly during mouse development, and specifically in the vomeronasal organ or olfactory bulb (*p*<10^−3^, EFDR = 0.05 entries; Figure 4A; Table S2). Genes expressed in three other central nervous system tissues (namely, frontal cortex, dorsal striatum and amygdala) also show associations with brain-expressed ncRNA loci, albeit at levels that are only marginal significant (*p*-value<10^−2^, EFDR = 0.53; Table S3). By way of contrast, ncRNA loci expressed in non-brain tissues have, as expected, a significant preference to be located next to protein-coding genes that are highly expressed outside of the central nervous system (Figure 4B; Table S2). These findings again point to functional interactions between genetically-linked pairs of non-coding and protein-coding transcripts.

Genetic interactions between adjacent coding and non-coding transcripts might be reflected in a preference for their transcription in *sense* (same) or *antisense* (opposite) directions. Indeed, brain-expressed ncRNA loci and their adjacent protein-coding genes strongly exhibit a preference for transcription in sense (73%, *p*<10^−10^); a similar, but weaker, significant tendency was observed for constrained ncRNAs expressed outside of the brain (56%, *p* = 0.01) (Table S1). The ncRNA we considered are transcribed from largely intergenic loci and are mainly full-length in sequence. Nevertheless, these biases in sense-transcription may be explained if their sequences are also contained within alternative transcripts from protein-coding gene loci. This possibility was explored, and eventually discounted, following investigation of twelve pairs of closely neighbouring non-coding and coding gene loci (see below). We were also able to discount a model involving a ‘rippling’ of transcription across neighbouring loci [22] (see Discussion).

### Experimentally validated transcriptional and temporal co-localisation of non-coding and protein-coding transcript pairs

**Figure 5:**
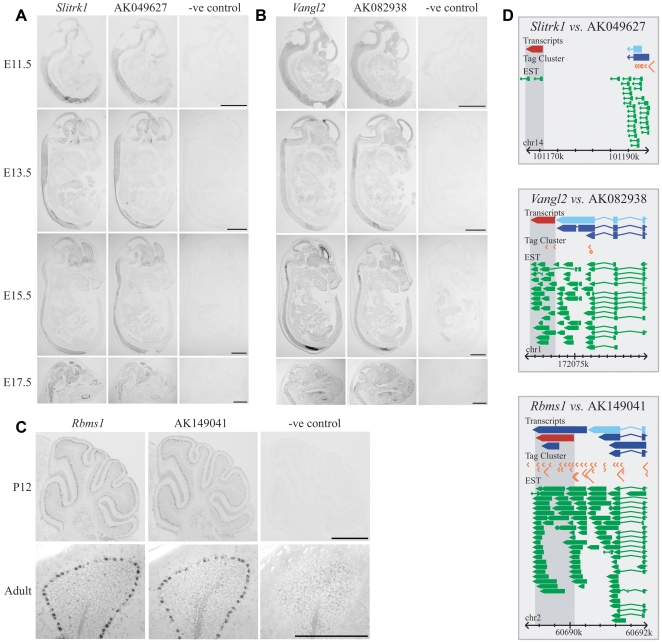
Developmental neuronal expression patterns of *Slitrk1*, *Vangl2*, and *Rbms1* overlap with those from ncRNAs transcribed from adjacent genomic sequence. Brightfield images of *in situ* hybridization from adjacent wild-type sections are shown. (A) *Slitrk1* and the ncRNA AK049627 (derived from an E12 spinal cord cDNA library) are expressed throughout mid/late embryonic development, with the specific co-expression in the brain and spinal column. (B) A similar pattern of co-expression in the CNS is observed for *Vangl2* and the adjacent ncRNA AK082938 (derived from an E12 spinal cord library). (C) AK149041 (isolated from a P2 sympathetic ganglion library) was expressed with the adjacent *Rbms1* gene at low levels in all major regions of the post-natal and adult brain (data not shown), although high levels of co-expression are observed in the developing Purkinje cell layer in the cerebellum from P12 to adulthood; higher magnification of the adult cerebellum shows that expression of both transcripts occurs in individual Purkinje cell bodies. The sense strand probe from the corresponding protein-coding gene is also shown. (A, B, C) Scale bars represent 2 mm in all cases. No expression information regarding any of these ncRNAs is currently available from the Allen Brain Atlas [23]. (D) The genomic landscape for each protein-coding (light blue) and non-coding (red) transcript pair is shown. Experimental evidence for transcription in the form of CAGE tag clusters (TC) (orange) [1],[18] and EST (green) data are also represented (as modified from the FANTOM3 Mouse Genomic Element Viewer (http://fantom32p.gsc.riken.jp/gev-f3/gbrowse/mm5): only unique transcripts and ESTs are shown). The size of a TC reflects the number of CAGE tags that are mapped to this region. A TC and its surrounding genomic sequence together can be considered a core promoter. It is evident that all three ncRNAs have further experimental support from ESTs (including those that are unspliced) and/or CAGE TCs (also listed in Table S4). AK082938 and AK149041 ncRNA transcripts are overlapped by ESTs and CAGE TCs that are derived from brain-associated tissues from adult and developing mice, whereas AK049627 has EST support from brain-associated tissues from developing mice.

Constrained long ncRNA loci thus exhibit preferences to be transcribed on the same strand as adjacent protein-coding genes that are expressed in similar tissues and that often function as transcription regulators. To test this model experimentally by *in situ* hybridisation, we selected 6 pairs of ncRNA and mRNA, transcribed from adjacent genomic loci, whose ncRNA transcripts were identified originally from embryonic or neonatal mouse brain libraries. These pairs were chosen essentially at random, except that they were required to be transcribed in the same orientation in order to test experimentally for read-through transcripts between coding and non-coding loci (see below). Experimental evidence for independent promoters for individual ncRNAs and genes was provided by CAGE tags (Figure 5). Note that because these experiments investigated expression at developmental time-points, relevant data from the Allen Brain Atlas are not available.

Across a range of embryonic and postnatal time-points, all 6 ncRNA and protein-coding gene pairs tested display overlapping expression patterns in the CNS (Figure 5A–5C and Figure S3). For example, co-expression of *Slitrk1* with AK049627 (Figure 5A), and *Vangl2* with AK082938 (Figure 5B), were maintained throughout mouse development, from E11.5 to E17.5. For the transcription factor *Zic4*, however, embryonic expression was highly localised to the spinal cord and regions of the forebrain, whereas the paired ncRNA was ubiquitously expressed (Figure S3). At postnatal time-points, *Rbms1* was co-expressed together with its paired ncRNA AK149041 at low levels throughout the brain, but most notably in the Purkinje cells of the cerebellum, from P12 (Figure 5C) to adulthood (data not shown). In addition, both *Meis1* and *Grik2* were expressed at very low levels at P12 apart from in the cerebellar granule cell layer; their respective ncRNAs were also only detectable in the same population of cells (Figure S3).

**Figure 6:**
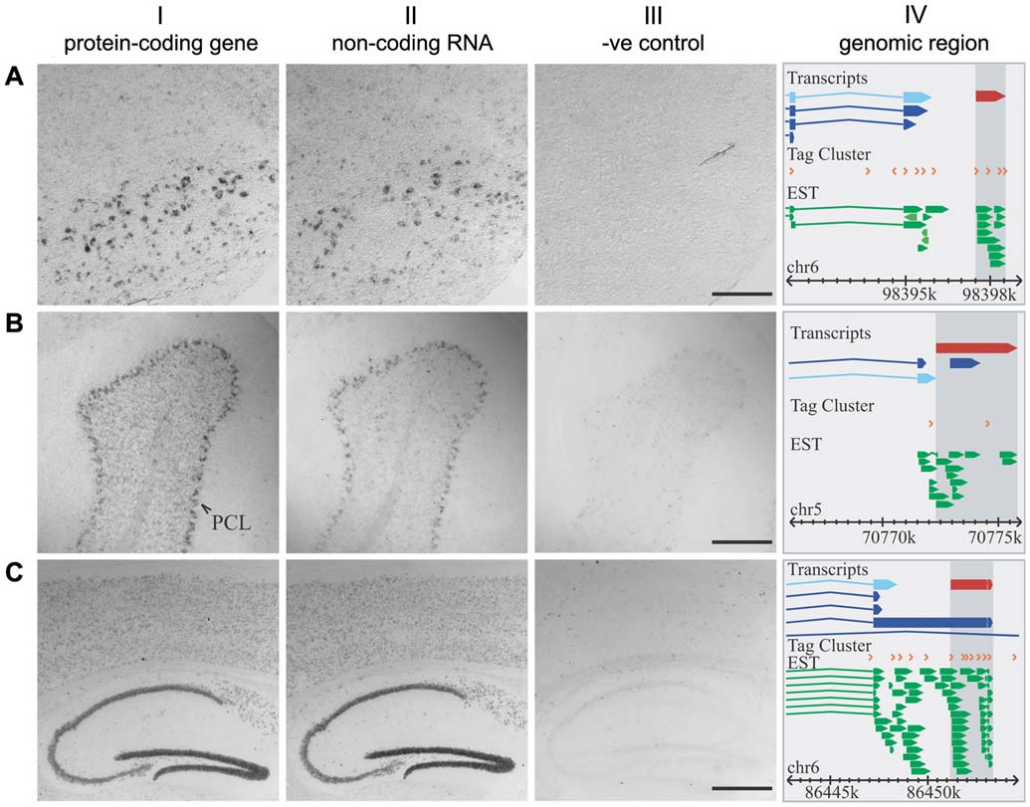
Adult brain expression patterns of *Mitf*, *Gabrb1*, and *Add2* overlap with those from ncRNAs transcribed from adjacent genomic sequence. Brightfield images of *in situ* hybridization from adjacent wild-type adult male 8-week old brain sections are shown. (A) Both *Mitf* (I) and AK018196 (II) were co-expressed at low levels throughout the brain including the olfactory bulb (data not shown) but also show high levels of expression in the facial nuclei of the medulla. (B) *Gabrb1* (I) and AK045528 (II) are co-expressed in most brain regions (data not shown), including specifically around the Purkinje cell layer of the cerebellum. (C) *Add2* (I) and AK013768 (II) are also expressed in all areas of the brain, but levels are substantially higher in the hippocampus in both cases. (A, B, C) The sense strand probe from the corresponding protein-coding gene is shown (III). Scale bars represent 0.25 mm (A, B III) and 0.5 mm (C III). No expression information regarding any of these ncRNAs is currently available from the Allen Brain Atlas [23]. Column IV represents the genomic landscape for each protein-coding (light blue) and non-coding (red) transcript pair. Experimental evidence for transcription in the form of CAGE tag clusters (TC) (orange) [1],[18] and EST (green) data are also represented (as modified from the FANTOM3 Mouse Genomic Element Viewer (http://fantom32p.gsc.riken.jp/gev-f3/gbrowse/mm5): only unique transcripts and ESTs are shown). The size of a TC reflects the number of CAGE tags that are mapped to this region. A TC and its surrounding genomic sequence together can be considered a core promoter. It is evident that all three ncRNAs have further experimental support from ESTs (including those that are unspliced) and CAGE TCs (also listed in Table S4). AK045528 and AK013768 ncRNA transcripts are overlapped by ESTs and CAGE TCs that are derived from brain-associated tissues from adult and developing mice, whereas AK018196 has support from adult mouse brain ESTs.

Similarly, at random, we chose an additional 6 protein-coding and non-coding pairs for which the ncRNA was initially identified in the brains of adult mice. Available data [23] also indicate expression for each of these 6 protein-coding genes within the specific sub-region of the adult brain from which its partner ncRNA transcript was originally derived. Of the 6 adult-expressed protein-coding gene partners, all were detectable in the brain by *in situ* hybridisation; of these, the expression patterns of 5 overlapped with those of their adjacent non-coding RNAs (Figure 6 and Figure S3).

Extensive evidence was available from CAGE tags that each long ncRNA we examined represented a transcript that was independent of the upstream protein-coding gene (Figure 5 and Figure 6; Table S3). Nevertheless, we decided to investigate whether any long cDNAs derive from transcriptional read-through of a single transcript spanning the 3′ UTR of a neighbouring protein-coding gene locus and the ncRNA locus. If so, this might explain our previous observations of co-expression and transcription in *sense*. We performed RT–PCR experiments for 8 ncRNAs whose intergenic distance to the closest protein-coding gene was less than 25 kb. Results showed that in all but one case no such read-through transcript could be identified from within the particular tissues and/or at the specific time-points used to generate the *in situ* hybridisation data (Figure S4).

Next, we used 5′ RACE experiments to confirm the transcription start sites that are expected from these ncRNAs' database sequences. Importantly, for this we obtained sequence only from the same brain tissue and at the specific developmental timepoint in which we had shown, by *in situ* hybridisation, expression of the relevant ncRNA. Using a method specific for full-length, capped mRNA species, products of the expected sizes and sequences were amplified for 11 of the 12 selected ncRNAs (Figure S4). The one exception in these 5′ RACE experiments (an exception, also, for the RT–PCR experiments) was the *Add2*/AK013768 pair; these experiments identified an *Add2* splice variant transcript with an extended 3′ UTR spanning the entire AK013768 ncRNA sequence. Indeed, this variant transcript (accession NM_008601) had been identified previously as being brain-specific [24] and thus represented a positive-control in our experiments. One ncRNA, AK162901, whose genomic locus lies adjacent to *Adr* could not be detected by RT–PCR, 5′ RACE or *in situ* hybridisation. Aside from these two examples, our data demonstrate that the overlapping *in situ* hybridisation patterns for 10 out of the 12 ncRNAs tested cannot be derived simply from 3′ UTR extensions of these protein-coding genes; instead, they represent independent transcriptional units that are expressed in the nervous system.

## Discussion

Our studies demonstrate strong and significant preferences for 659 constrained long and intergenic ncRNAs to be transcribed in proximity to transcriptional regulator genes, and to be enriched in predicted RNA secondary structures. Moreover, brain-expressed ncRNAs were shown to be transcribed preferentially near to brain-expressed protein-coding genes. We investigated whether this preference arose simply from ncRNA and coding transcripts sharing exons in splice variants (“transcriptional read-through”), yet found no evidence that this occurs for 11 of the 12 examples we investigated; the single exception validated a previously identified alternative transcript. In Text S1 we show that the magnitude of differential protein-coding gene expression across tissues is insufficient to explain the significant tendency for 239 brain-expressed ncRNAs to be transcribed adjacent to brain-expressed protein-coding genes; in fact, transcriptional read-through would in some cases predict tendencies opposite to our observations. Moreover, aside from the said single extension of a protein-coding 3′ UTR into a ncRNA locus, we find no cDNA evidence for transcriptional read-through. Instead, there is abundant evidence from CAGE tag data for transcription start sites that correspond to ncRNA cDNA sequences.

Our findings on intergenic ncRNA loci complement and extend those from other studies that focused on ncRNA loci that overlap protein-coding genes [2],[3]. One of these studies showed that 64% of ncRNAs are expressed in the brains of 56 day-old mice [2]. Our focus on a lower number of ncRNAs allowed comparison of ncRNA and gene expression profiles across a range of developmental stages, and was able to demonstrate expression of a similar proportion (10 of 12 assayed) of long ncRNAs in the mouse brain.

### Properties indicative of ncRNA function

Instead of ‘transcriptional noise’, the enrichment of predicted RNA secondary structures in constrained ncRNAs (Figure 1B), the comparable expression levels of presumably stable ncRNA and protein-coding transcripts (Figure 5, Figure 6), and ncRNAs' increasing constraint moving away from protein-coding sequence [15] all point to the RNA sequences themselves conveying diverse regulatory functions. Previously, we also demonstrated that splice site dinucleotides of mouse long ncRNAs are better conserved to human and to rat than expected by chance [15]. An example of canonical GT-AG splice site consensus sequence motifs that are conserved to human and to rat lies within the 5′ of mouse AK090266, a long ncRNA locus transcribed bidirectionally with *Cited1*, a regulator of CBP/p300-dependent transcriptional responses. Long ncRNAs with predicted RNA secondary structures may be processed to form smaller functional RNAs. Evidence for widespread processing of long ncRNAs remains scant [25],[26] although some of the set we examined (including AK080813, for example, which harbours the mmu-mir-568 microRNA sequence) may yet be shown to be precursors of smaller *trans*-acting molecules.

The annotated functions of the adjacent protein-coding genes are consistent with the general functional biases observed among non-coding and coding transcript pairs. Some of the genes assayed encode known transcriptional regulators (*Rbms1*, *Mitf, Zic4*), some possess functions in the developing CNS (*Vangl2*, *Slitrk1*, *Gabrb1, Zic4*) and some, when disrupted, are associated with disease (*Vangl2*, *Slitrk1*, *Mitf*, *Gabrb1*, *Add2, Zic4*) [27]–[34]. Given their sequence conservation and predicted RNA secondary structures, it is likely that mutations within constrained long ncRNAs will be deleterious, although whether such deleterious variants would often manifest as observable phenotypes remains to be determined.

We have conservatively identified 659 constrained long and intergenic ncRNAs that appear the most likely to be functional, as opposed to being transcriptional noise. Nevertheless, many ncRNA sequences for which we could not detect constraint may yet be functional. For example, *Evf2*, which is known to act as a *Dlx-2* transcriptional coactivator [9], and *Neat1* (AK159400), which is essential for the structure of nuclear paraspeckles [35], are each not considered as being under constraint in our analysis. Our inability to detect constraint in some functional ncRNA sequences is, in part, owing to the low amount of functional sequence within them: the average proportion of a ncRNA locus that can be identified as being under constraint is approximately 5% [15]. In addition, because we are estimating constraint between mouse and human sequence, lineage-specific ncRNAs such as mouse *B2* [36],[37] will be overlooked by our approach.

### Potential ncRNA cis-regulatory mechanisms

Co-expression and genomic co-localisation of these non-coding and coding locus pairs is consistent with their transcriptional co-regulation in *cis*. Our studies were not intended to investigate the genetic action of non-coding gene loci in *trans* or over long physical distances, although some long ncRNAs may act in *trans* if their predicted secondary structures are the targets of transcriptional regulatory RNA-binding proteins. Instead, we focused our attention on *cis*-regulatory coding and noncoding gene partners because the mechanisms of long ncRNA loci, when known, often are exerted over short-ranges (reviewed in [4]), and because many such loci lie in very close proximity to protein-coding genes [15],[38].

These *cis*-regulatory long ncRNAs, as for other molecular types such as proteins or ‘housekeeping’ RNAs, are likely to convey a broad spectrum of molecular functions. For some, it will be their transcription driving chromatin remodelling that regulates the transcription of neighbouring (and not necessarily adjacent) protein-coding genes [6],[39], perhaps by facilitating access to enhancers and promoters for transcriptional machinery molecules. This is of particular relevance to transcription factor genes since their genomic loci and flanking regions tend to be replete in conserved noncoding sequence [40],[41]. In other cases, long ncRNAs may ‘coat’ double-stranded DNA as it appears to do in epigenetic gene silencing, or it may suppress transcription of the neighbouring protein-coding gene by transcriptional interference (reviewed in [4]). These three possibilities are consistent with stronger sequence conservation within these ncRNAs' promoters than in their transcripts' sequences [1],[15]. Long ncRNAs may also bind DNA-bound factors that expedite or suppress transcription of adjacent loci.

One possibility that we considered initially, and then discarded, is that transcription of these ncRNAs is an inconsequential result of neighbouring ‘intermediate-early’ protein-coding genes (IEGs) being transcribed [22]. However, long ncRNA loci in our data set are depleted, rather than enriched, within IEGs and their immediate 100 kb up- and downstream flanking sequence (no overlap; *p* = 0.57 for enrichment; IEGs from [22]). We considered one further explanation of the close vicinity of long ncRNA and transcription factor gene loci. This supposes that the ncRNA promoter is one of the downstream targets of the transcription factor, perhaps participating in a feedback or feedforward loop thereby regulating the level of transcription factor expression. Nevertheless, our observations that transcription factor genes are expressed at higher levels and in a greater range of tissues than their genomically neighbouring ncRNA loci argue that it is their promoters, and not those of the long ncRNAs, that are the downstream targets.

The well-characterized regulatory ncRNAs to date convey a broad variety of functional roles. Thus, the molecular mechanisms of the long ncRNAs presented here are not expected to proceed only in one regulatory model. Nevertheless, our findings are consistent with mechanisms by which long ncRNA loci provide subtle and tissue-specific regulatory control over neighbouring protein-coding gene loci. This is because these long ncRNA loci tend to be transcribed at low levels and in restricted numbers of tissues, whilst their neighbouring protein-coding loci are mainly transcribed at higher levels and more broadly, in greater numbers of tissues.

The importance of our findings concerns the insights they provide into the extensive, yet unannotated, mammalian transcriptome. In the midst of the large amount of the un-annotated transcriptome, these insights allow an objective prioritization of long ncRNA loci that are likely to regulate expression of adjacent protein-coding transcriptional regulators in the brain. They will thus be critical in the design of experiments seeking to investigate the large number of non-coding transcripts, reported by the ENCODE project [42] and by others [1], [43]–[46], whose functions remain virtually all unknown. The ncRNA transcripts, and annotations relating to expression, constraint, copy number variation and predicted RNA secondary structures, are provided in Table S5 and Table S6.

## Materials and Methods

### Data sets

We considered a set of 3,122 long intergenic ncRNAs derived from mouse cDNA libraries [1],[47]. These ncRNAs have been purged of those containing long open-reading frames, they are virtually exclusively located outside of protein-coding gene models (3% overlap such models but are on the complementary strand) and, as shown elsewhere, they are enriched in sequence that is constrained with respect to nucleotide substitution and to insertion or deletion [15]. After removing 62 overlapping ncRNAs (see below), this set was further divided according to the transcript's spatiotemporal expression and the degree of constraint on nucleotide substitutions. Specifically, ncRNAs were divided into those derived from brain tissues and non-brain tissues, and further into those showing (or not showing) evidence of constraint in mouse-human comparisons (see below). ncRNAs derived from multiple tissues such as head and whole body (469) were not considered further. Overall, 1,932 ncRNAs were classified as non-constrained; these include transcripts whose evolution is indistinguishable from neutrality, as well as mouse transcripts with insufficient numbers of aligned positions (<500 bp), when compared to orthologous human sequence, to allow reliable estimation of evolutionary rates. Of these non-constrained ncRNAs, 579 are known to be expressed in the brain. Overall, 255/659 of constrained and 523/1,932 of non-constrained transcripts were supported by CAGE tag clusters (TCs) [1],[18] lying within 100 bp of their transcriptional start site. ncRNA data sets are listed according to constraint in Table S5.

To determine tissue specificity of protein-coding genes we employed the relative entropy (RE; Kullback-Leibler distance) measure based on the distribution of CAGE tags from different tissues [21]. Protein-coding genes were selected whose tag cluster contained more than 30 CAGE tags. The Kolmogorov-Smirnov test was used to investigate whether two RE data sets may reasonably be assumed to sample the same distribution.

### ncRNAs derived from different tissues and developmental stages

Each ncRNA was assigned a tissue and a developmental stage according to information present in its cDNA library entry [1],[47]. In 62 instances, multiple ncRNAs mapped to the same genomic locus. In all but three of these cases the multiple ncRNAs were derived from a single tissue. In these three exceptional cases, all ncRNAs were derived from non-brain tissues. By excluding ncRNA loci expressed in head and whole body cDNA libraries, we further classified ncRNAs into two tissue classes and two developmental stage classes: (*i*) those expressed in one of 15 CNS tissues (brain, cerebellum, corpora quadrigemina, corpus striatum, cortex, diencephalon, hippocampus, hypothalamus, medulla oblongata, olfactory brain, pituitary gland, spinal cord, spinal ganglion, sympathetic ganglion and visual cortex) defined as *brain-derived* ncRNAs, (*ii*) those expressed in one or more of 45 different tissues from outside the CNS, (*iii*) those expressed during embryonal or neonatal development, and (*iv*) those expressed in adult mice.

### Estimation of nucleotide substitution rates in non-coding sequence

Nucleotide substitution rates between orthologous mouse-human aligned sequences were estimated and compared with local rates estimated from local ancestral repeats (ARs) as described elsewhere [15]. To accurately estimate substitution rates, we only considered ncRNAs' alignments exceeding 500 bp in length. Local ARs had to fulfil two criteria as described in [15], *viz*. (*i*) no overlap with its local ncRNA, and (*ii*) minimal length of 100 bp, and additionally: (*iii*) no overlap with indel-purified segments (IPSs) (identified at a false discovery rate (FDR) of 10% [48] in order to exclude any selectively purified sequence), and (*iv*) a location within 500 kb up- and downstream of the ncRNA neighbouring region to ensure a similar local mutation rate. To determine whether a specific ncRNA exhibits a significantly suppressed substitution rate (*d_RNA_*) compared to the expectation under neutrality, we estimated the local neutral rate by randomly sampling local ARs in 1,000 iterations. Local ARs that fulfilled the above criteria were selected randomly and concatenated until the total ungapped alignment length of these AR sequences exactly matched the length of the aligned fraction of the ncRNA sequence. Subsequently, the average substitution rate (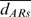) of these concatenated AR sequences was estimated. A ncRNA was considered to have been subject to a significant degree of purifying selection if fewer than 25 of the 1,000 d_ARs_ values were less than *d_RNA_* (*i.e. p*<0.025). Use of the mean *d_AR_*_s_ value was justified owing to these values being normally distributed (data not shown). In total, 659 ncRNAs derived from brain or elsewhere were inferred to have been subject to significant levels of purifying selection, with a false discovery rate (FDR) less than 0.025 (16 expected cases).

### Genome-wide association procedure controlling for G+C–content biases

To assess whether long ncRNA segments *S* are significantly associated with functional annotations among genomic elements *E* within a subset of the genome *I*, while accounting for any G+C–content biases and chromosome-specific biases, we applied a randomization procedure [15]. This compares, within *I*, a defined set of genomic segments *S* against multiple randomized sets of segments *S′*, which are chosen to have the same genomic overlap within G+C-stratified subsets of *I* and within each chromosome, and to have a matched length distribution. The set *S* and sets *S′* are compared with respect to their overlap with a specified fixed set of intervals *E* that are associated with a particular annotation. To obtain accurate *p*-values, simulation runs were performed 10,000 or 100,000 times. This procedure was applied to five annotation sets *E*: (*i*) indel-purified segments identified at a FDR of 10% [48]; (*ii*) PhastCons multispecies' conserved elements [49]; (*iii*) EvoFold predictions of RNA secondary structure [50]; (*iv*) non-overlapping human copy number variants (CNVs) [51] and (*v*) non-overlapping human segmental duplications [52]. *I* was defined as intergenic sequences located between ENSEMBL-annotated protein-coding genes [53]. To account for the ascertainment bias in case (*iii*), resulting from EvoFold searching for RNA structure only within conserved sequence, we restricted *I* to those intergenic sequences that are multiply aligned to genomic sequences of five or more vertebrate species in the 8-way MultiZ alignments [54], and exhibit overlap with PhastCons multispecies conserved elements; this filtering procedure is similar to that used in the EvoFold pipeline (Petersen JS, pers. comm.). If not otherwise stated, data were obtained from the UCSC Genome Browser Database [55]. Association studies (*i*) to (*v*) that were significant resulted in *p*-values<2×10^−4^ and experimental false discovery rate (EFDR) values<10^−3^.

### Functional and expression association

We assessed whether the functional categories of those protein-coding genes that are nearest to the genomic loci from where the ncRNAs are transcribed sample the functions of all genes randomly. For this, we considered Gene Ontology (GO) [56] annotations associated with these nearest protein-coding Known Genes (based on UniProt, RefSeq and GenBank mRNA) [55]. To test for expression associations, we used GNF Gene Expression Atlas data of all 61 non-cancer mouse tissues [57] by mapping the Locus Link identifier to Known Genes. A gene was classified as being highly expressed in a tissue if its expression exceeded the median calculated across these 61 tissues by 8-fold or more. We assigned a non-coding transcript to its closest known protein-coding gene *i* if it overlapped with this protein-coding gene's “territory”, defined as nucleotides that are closer to gene *i* than they are to the most proximal up- and down-stream protein-coding genes *i+1* and *i−1*. The territory of overlapping protein-coding genes constitutes the maximal region both genes occupy until the mid-distance to the next most proximal genes. The sampling procedure outlined above ensures that systematic variations in territory size, resulting from variations in gene density, will not result in biased outcomes from the association test (although the power to detect these associations will be affected). To discount significant GO and GNF associations for annotations that occur at low frequency, which otherwise would lead to high FDRs, we only considered GO and GNF terms each with an associated territory covering at least 1% of the genome (resulting at *p*<10^−3^ in EFDR = 0.08 and EFDR = 0.05, respectively). By applying these significance thresholds, we tested whether protein-coding genes of a particular GO category are enriched close to ncRNAs derived from different classes (see above). In particular, when considering constrained and brain-derived ncRNAs that are expressed (*i*) in adult mice or (*ii*) during mouse development, we found significant associations for (*ii*) but not for (*i*). It is notable that distributions of distances from a ncRNA to its closest protein-coding gene for these two classes are not significantly different (*p* = 0.2, Kolmogorov-Smirnov test).

### Strand bias

To determine whether there is a preference for ncRNAs to be transcribed in the same (sense) or opposite (antisense) direction relative to their neighbouring protein-coding genes, we used the defined genomic coordinates of Known Genes as described above. ncRNAs that overlap two gene territories or that coincide with a territory containing overlapping genes transcribed on both strands were discarded. We separately counted those ncRNAs transcribed in sense 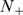, and those in antisense 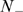, orientations, and tested the null hypothesis that the directions of transcription of a ncRNA transcript and its neighbouring protein-coding gene are not associated (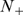 and 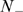 binomially distributed with *p* = 0.5). The high 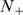 and 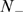 counts justify the use of a normal approximation to the binomial distribution.

### *In situ* hybridisation

Fragments of each target of approximately 400 bp were amplified by RT–PCR from mouse whole brain cDNA or by PCR from genomic DNA and cloned into pCR4-TOPO (Invitrogen); primer sequences are available on request from the authors. Probes for the protein-coding genes were designed to represent transcripts from all annotated splice variants. Dioxygenin-labeled riboprobes were synthesized using the appropriate RNA polymerase for both the anti-sense and sense strands. Mouse brain and whole embryos were frozen in OCT (VWR) on dry ice, and 10 µm parasagittal cryosections were cut and mounted on positively charged slides. Adjacent sections were hybridized to probes for each protein-coding gene and corresponding ncRNA with sense strand probes used as a negative control in all cases. Hybridizations and signal development were performed as previously described [58], with all slides developed for 24 hours prior to mounting and microscopy.

### RT–PCR and 5′ RACE expression analysis of protein-coding and non-coding transcripts

For both RT–PCR and 5′ RACE experiments, tissue from C57BL/6 mice was obtained from wild-type 56 day old adults or from the developmental stage at which expression of the ncRNA had been observed by *in situ* hybridisation. Total RNA was purified using the RNeasy Midi kit (Qiagen) and subsequently DNAse treated as recommended. For RT–PCR, cDNA was synthesized using Expand Reverse Transcriptase (Roche) and amplified with 35 cycles using Expand Hi-Fidelity Polymerase (Roche). 5′ RACE was performed using a RNA Ligase-Mediated RACE (RLM-RACE) method. Briefly, total RNA was de-phosphorylated with alkaline phosphatase to select for full-length transcripts, followed by treatment with tobacco acid pyrophosphatase and ligation of a RACE adaptor primer (5′ GCUGAUGGCGAUGAAUGAACACUGCGUUUGCUGGCUUUGAUGAAA) to the newly decapped mRNA. After reverse transcription with Expand Reverse Transcriptase (Roche), cap-specific products were amplified with Expand Long Template polymerase (Roche) using a reverse primer approximately 350 bp from the predicted transcription start site of each ncRNA and a forward primer specific for the RACE adaptor (5′ GCTGATGGCGATGAATGAACACTG). An aliquot of each reaction was then used as a template with a nested ncRNA and nested forward primer (5′ GAACACTGCGTTTGCTGGCTTTGATG). Amplified products were cloned into the pCR4-TOPO or pCR-XL-TOPO TA-cloning vectors (Invitrogen) and sequenced. Optimal amplification conditions were determined by adjusting the annealing temperature in all cases.

## Supporting Information

Figure S1 Constrained ncRNAs (AK082637 (A), AK082142 (B), and AK032637 (C)) that are expressed in the cerebellum during mouse development and that contain predicted RNA secondary structures. For each ncRNA, its genomic region, its overlapping EvoFold predicted segments (EvoFold track, shown in red) and its evolutionary conservation in mouse, rat, human, dog, and chicken (based on phastCons scores, UCSC genome browser representation (Karolchik et al., 2008) are shown (left panels). RNA secondary structures, predicted using RNAalifold (Hofacker et al., 2002), are also shown (right panels). RNAalifold's notation indicates paired positions with consistent mutations using circles around the varying position, compensatory mutations using circles around both pairing partners, and inconsistent mutations by gray, instead of black, lettering. Karolchik, D, Kuhn RM, Baertsch R, Barber GP, Clawson H, et al., (2008) The UCSC Genome Browser Database: 2008 update. Nucleic Acids Res, 36: D773-9. Hofacker, IL, Fekete M, Stadler PF, (2002) Secondary structure prediction for aligned RNA sequences. J Mol Biol, 319: 1059-66.
(2.17 MB TIF)

Figure S2 Dinucleotide distribution analysis of CAGE tag starting sites with varying amounts of CAGE tag support for long ncRNAs (panels A and B) and their adjacent protein-coding transcripts (panels C and D), partitioned according to whether the long ncRNA is expressed in the brain (panels A and C) or elsewhere (panels B and D). Shown are the different [−1, +1] dinucleotides relative to each CAGE tag starting sites in the data set (note that the −1 nucleotide is not part of the sequenced tag). These cases were subdivided according to the numbers of tags supporting the CAGE tag starting sites (1,2,3 to 9 tags, and >9 tags).
(0.03 MB DOCX)

Figure S3 Co-expression of further protein-coding/non-coding RNA transcript pairs in the developing (Panels A, B, C) and adult (Panels D, E, F) CNS. Brightfield images of *in situ* hybridization from adjacent wild-type sections are shown. (A) Expression of the ncRNA AK082989 appeared ubiquitous in an E13.5 embryo, although *Zic4*, the adjacent protein coding gene, showed a highly specific pattern of expression in the spinal cord and forebrain at the same time-point, as was described previously (Gaston-Massuet et al., 2005). (B) At P12, *Meis1* is only expressed above background levels in the developing cerebellar granule cell layer, where the ncRNA AK042766 is also found expressed. (C) *Grik2*, however, is expressed ubiquitously in the brain, although the adjacent ncRNA AK047467 is only found at low levels in the cerebellar granule cell layer at P12. (D) Both *Hip2* and its paired ncRNA, AK045758, are expressed at high levels in the cortex and the hippocampus. (E) *Eif2c3* is ubiquitously expressed in the brain, as is the genomically adjacent transcribed ncRNA, AK047638. (F) *Adr* also shows a ubiquitous expression pattern, although expression of its paired ncRNA, AK162901, is not detected in the adult brain, consistent with the RT-PCR results (Figure S3). In all cases, the sense strand negative control probe failed to show specific staining (data not shown). Gaston-Massuet, C, Henderson DJ, Greene ND, Copp AJ, (2005) Zic4, a zinc-finger transcription factor, is expressed in the developing mouse nervous system. Dev Dyn, 233: 1110-5.
(3.43 MB TIF)

Figure S4 RT-PCR and 5′ RACE analysis of protein-coding and non-coding transcripts. (A) Total RNA was purified from the tissues and the developmental time-points indicated. RT-PCR was performed using primers spanning from the 3′ UTR of the protein-coding gene to the adjacent ncRNA genomic sequence. Control amplification using the same primer pairs from genomic DNA (gDNA) and a reaction containing no reverse transcriptase (-RT) is also shown. Importantly, RT-PCR of each protein-coding gene and ncRNA was performed from the same tissue. Apart from *Add2*/AK013768, no evidence for read-through from the 3′ UTR to the ncRNA was observed that would account for the *in situ* hybridisation results obtained (Figure 5, Figure 6). (B) 5′ RACE products of all 12 ncRNAs analysed in this study (adjacent pc genes are indicated in brackets). Total RNA was purified from the tissue corresponding to the *in situ* hybridisation data: adult brain (AK018196 - AK162901), P12 cerebellum (AK149041, AK042766 and AK047467) and E13.5 brain (AK082938, AK049627 and AK082969). In these reactions, a nested reverse primer approximately 300 bp from the predicted ncRNA transcription start site and a nested forward primer specific for the cap-ligated RACE anchor primer was used. A reaction containing no reverse transcriptase (-RT) is also shown for each primer pair. RACE reactions containing no TAP enzyme showed no amplification products (data not shown).
(1.16 MB TIF)

Table S1 Brain-expressed ncRNAs are more likely to be constrained than ncRNAs expressed elsewhere (χ^2^-test, *p* = 3×10^−3^). This observed bias is independent of the lengths of these constrained ncRNAs since the length distributions of brain- and non-brain-expressed ncRNAs are indistinguishable (*p* = 0.4, Kolmogorov-Smirnov test). Transcripts classified as constrained or non-constrained were divided further into those transcribed in the same (*sense*) or opposite (*antisense*) direction relative to the transcriptional orientation of the most proximal protein-coding gene. Cases where a ncRNA is located near to protein-coding genes that are transcribed on both strands have been excluded. An asterisk (*) indicates a significant association with the direction of transcription of the proximal annotated protein-coding gene (see Materials and Methods). Non-constrained, brain-expressed ncRNAs show no directional preference, whereas non-brain-expressed ncRNAs show a small but significant bias in the opposite orientation (54% transcribed in antisense, p = 6×10^−3^).
(0.03 MB XLS)

Table S2 Constrained ncRNAs that are expressed in brain or in nonbrain tissues during development show a significant tendency to lie adjacent to proteincoding genes that are highly expressed in specific tissues (p<10^−3^; EFDR<0.04). Shown is the significant over-representation of ncRNAs in proximity to protein-coding genes that are expressed in these tissues as a result of the observed densities when compared to expected densities on randomly sampled G+C matched sequences; also shown are the lower and upper confidence intervals (CIs) at the 95% level and the standard deviation.
(0.02 MB XLS)

Table S3 Brain-expressed and constrained ncRNAs show a tendency to be transcribed near to protein-coding genes expressed in brain tissues. Shown are significant (p-value<10^−2^, EFDR = 0.53) and non-significant (highlighted in grey) enrichments. The observed densities of ncRNAs transcribed in proximity to protein-coding genes expressed in particular tissues have been compared to expected densities from randomly sampled G+C matched sequences (see Materials and Methods). Also shown are lower and upper confidence intervals (CIs) at the 95% level, and standard deviations (StdDev). Terms highlighted in bold correspond to results shown in Figure 4 (*p*-value<10^−3^, EFDR = 0.05).
(0.02 MB XLS)

Table S4 Experimental EST and CAGE TC (tag cluster) support for six non-coding transcripts (AK018196, AK045528, AK013768, AK149041, AK082938, AK049627) for which *in situ* hybridizations (ISHs) were performed (see Figure 5, Figure 6). Each of the six brain-derived and evolutionarily constrained ncRNA transcripts was further investigated for additional experimental evidence in the form of ESTs and CAGE TCs and the results are summarized in separate tables. For each EST and CAGE TC, its accession code, coordinates, strand, tissue type and stage are reported, and additionally for each EST its position (5′ or 3′) relative to the ncRNA is shown.
(0.08 MB XLS)

Table S5 ncRNA data sets used in this study: evolutionary and functional properties. The four sets contain ncRNAs that are (*i*) constrained and derived from brain-associated tissues, (*ii*) constrained and derived from tissues outside the CNS, (*iii*) non-constrained and derived from brain-associated tissues and (*iv*) non-constrained and derived from tissues outside the CNS. Each ncRNA is represented by its (*i*) accession code, (*ii*) genome coordinates (assembly mm5), (*iii*) strand information and (*iv*) whether it overlaps with: 1. EvoFold predictions of RNA secondary structure (EvoFold), 2. human copy number variants (CNVs), 3. segmental duplications (SDs), 4. PhastCons multispecies conserved elements (MCSs), and 5. indelpurified segments (IPSs). Overlap is indicated by the integer 1, lack of overlap by 0.
(0.35 MB XLS)

Table S6 ncRNA data sets used in this study: accession codes of all ncRNAs in these four data sets. The four sets contain ncRNAs that are (*i*) constrained and derived from brain-associated tissues, (*ii*) constrained and derived from tissues outside the CNS, (*iii*) non-constrained and derived from brain-associated tissues and (*iv*) non-constrained and derived from tissues outside the CNS. In particular, the two unconstrained data sets are listed in their entireties since in Table S5 only those that are homologous to human sequence are shown.
(0.16 MB XLS)

Text S1 Functional associations and transcript read-through.
(0.03 MB DOC)
