# Supplementary figures and images for: Genomic and Transcriptional Co-Localization of Protein-Coding and Long Non-Coding RNA Pairs in the Developing Brain
Source: PLoS Genet. 2009 Aug 21;5(8):e1000617. doi: 10.1371/journal.pgen.1000617 (PMC2722021; doi:10.1371/journal.pgen.1000617)

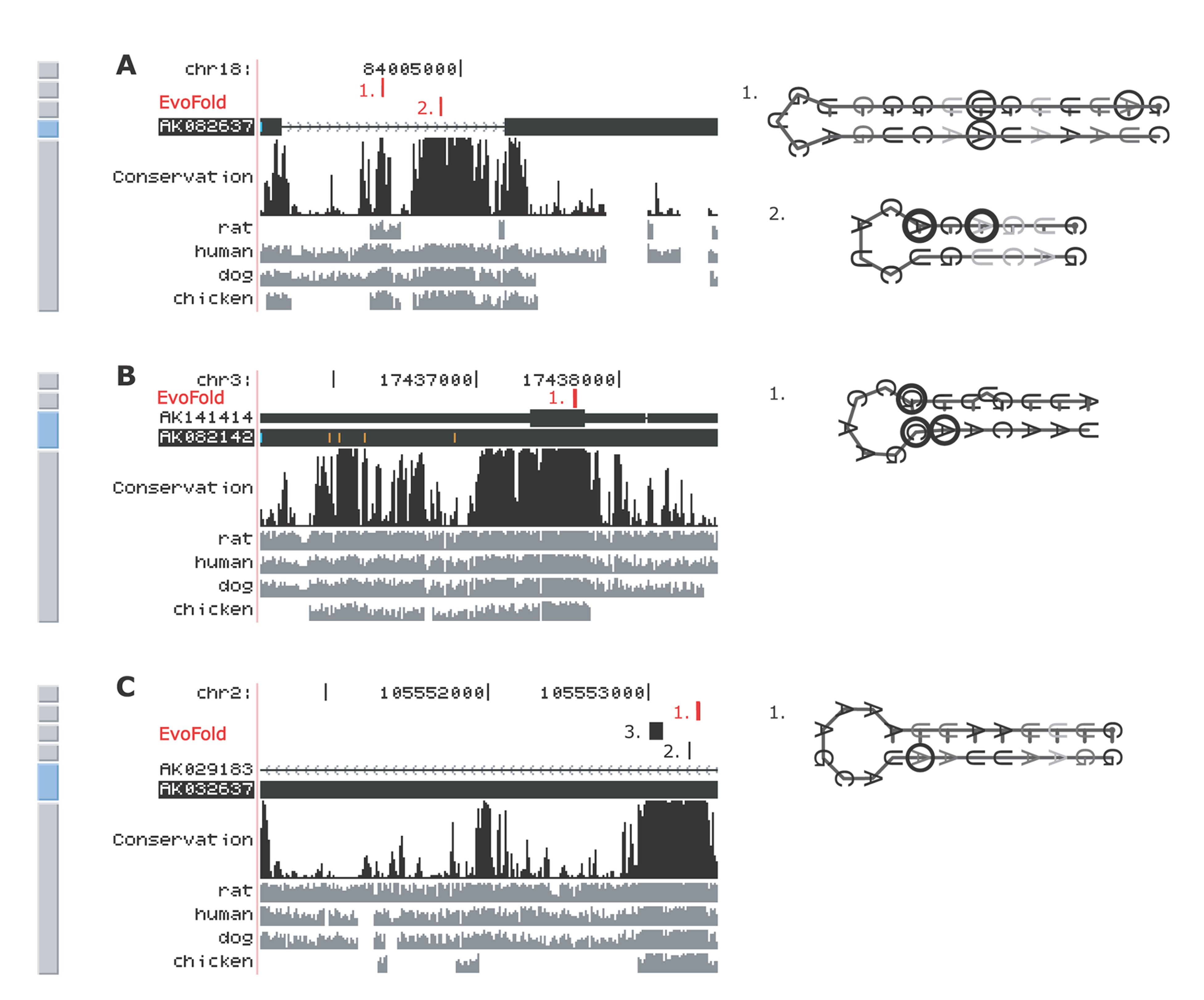

Supplement: Figure S1 — Constrained ncRNAs (AK082637 (A), AK082142 (B), and AK032637 (C)) that are expressed in the cerebellum during mouse development and that contain predicted RNA secondary structures. For each ncRNA, its genomic region, its overlapping EvoFold predicted segments (EvoFold track, shown in red) and its evolutionary conservation in mouse, rat, human, dog, and chicken (based on phastCons scores, UCSC genome browser representation (Karolchik et al., 2008) are shown (left panels). RNA secondary structures, predicted using RNAalifold (Hofacker et al., 2002), are also shown (right panels). RNAalifold's notation indicates paired positions with consistent mutations using circles around the varying position, compensatory mutations using circles around both pairing partners, and inconsistent mutations by gray, instead of black, lettering. Karolchik, D, Kuhn RM, Baertsch R, Barber GP, Clawson H, et al., (2008) The UCSC Genome Browser Database: 2008 update. Nucleic Acids Res, 36: D773-9. Hofacker, IL, Fekete M, Stadler PF, (2002) Secondary structure prediction for aligned RNA sequences. J Mol Biol, 319: 1059-66. (2.17 MB TIF) [file pgen.1000617.s001.tif]

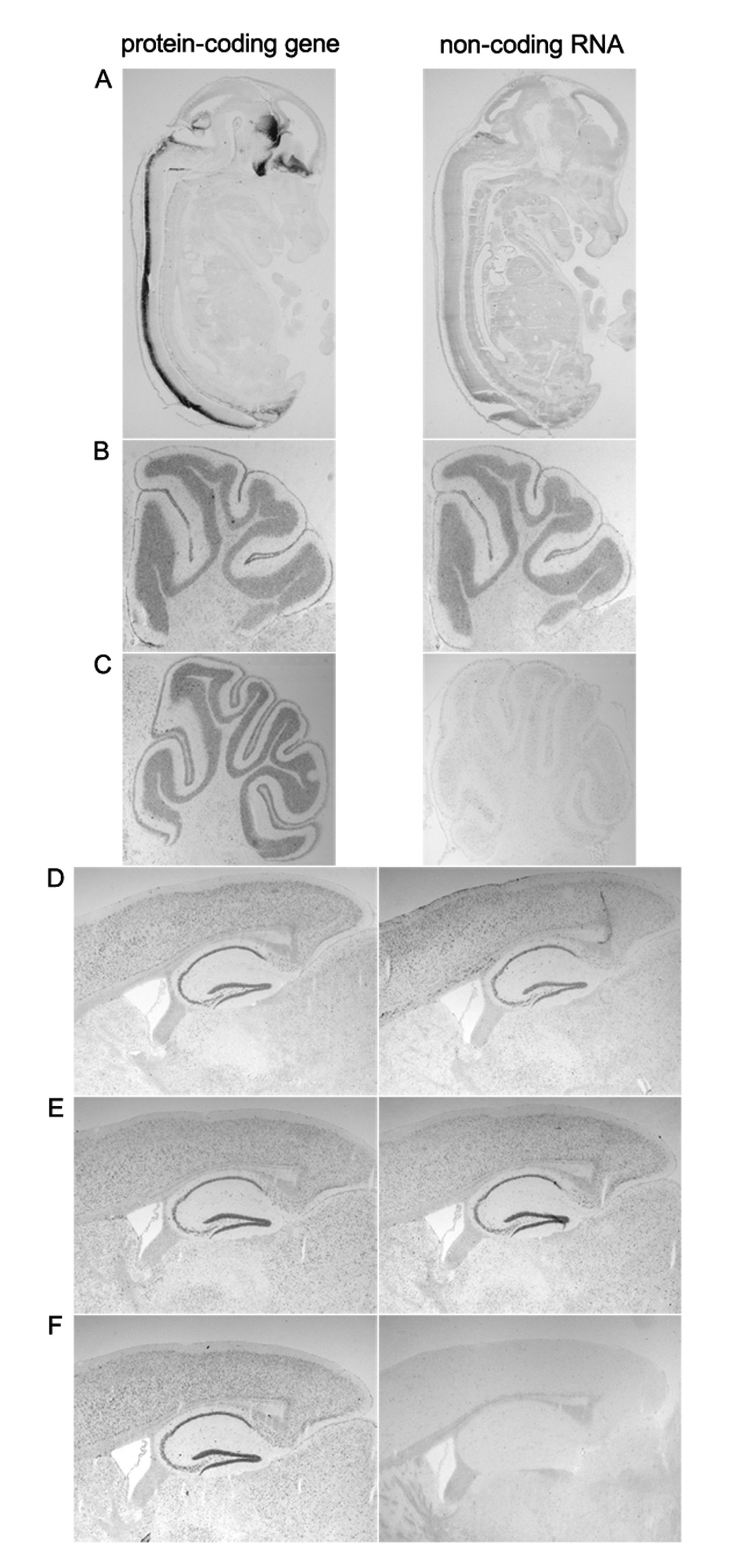

Supplement: Figure S3 — Co-expression of further protein-coding/non-coding RNA transcript pairs in the developing (Panels A, B, C) and adult (Panels D, E, F) CNS. Brightfield images of in situ hybridization from adjacent wild-type sections are shown. (A) Expression of the ncRNA AK082989 appeared ubiquitous in an E13.5 embryo, although Zic4, the adjacent protein coding gene, showed a highly specific pattern of expression in the spinal cord and forebrain at the same time-point, as was described previously (Gaston-Massuet et al., 2005). (B) At P12, Meis1 is only expressed above background levels in the developing cerebellar granule cell layer, where the ncRNA AK042766 is also found expressed. (C) Grik2, however, is expressed ubiquitously in the brain, although the adjacent ncRNA AK047467 is only found at low levels in the cerebellar granule cell layer at P12. (D) Both Hip2 and its paired ncRNA, AK045758, are expressed at high levels in the cortex and the hippocampus. (E) Eif2c3 is ubiquitously expressed in the brain, as is the genomically adjacent transcribed ncRNA, AK047638. (F) Adr also shows a ubiquitous expression pattern, although expression of its paired ncRNA, AK162901, is not detected in the adult brain, consistent with the RT-PCR results (Figure S3). In all cases, the sense strand negative control probe failed to show specific staining (data not shown). Gaston-Massuet, C, Henderson DJ, Greene ND, Copp AJ, (2005) Zic4, a zinc-finger transcription factor, is expressed in the developing mouse nervous system. Dev Dyn, 233: 1110-5. (3.43 MB TIF) [file pgen.1000617.s003.tif]

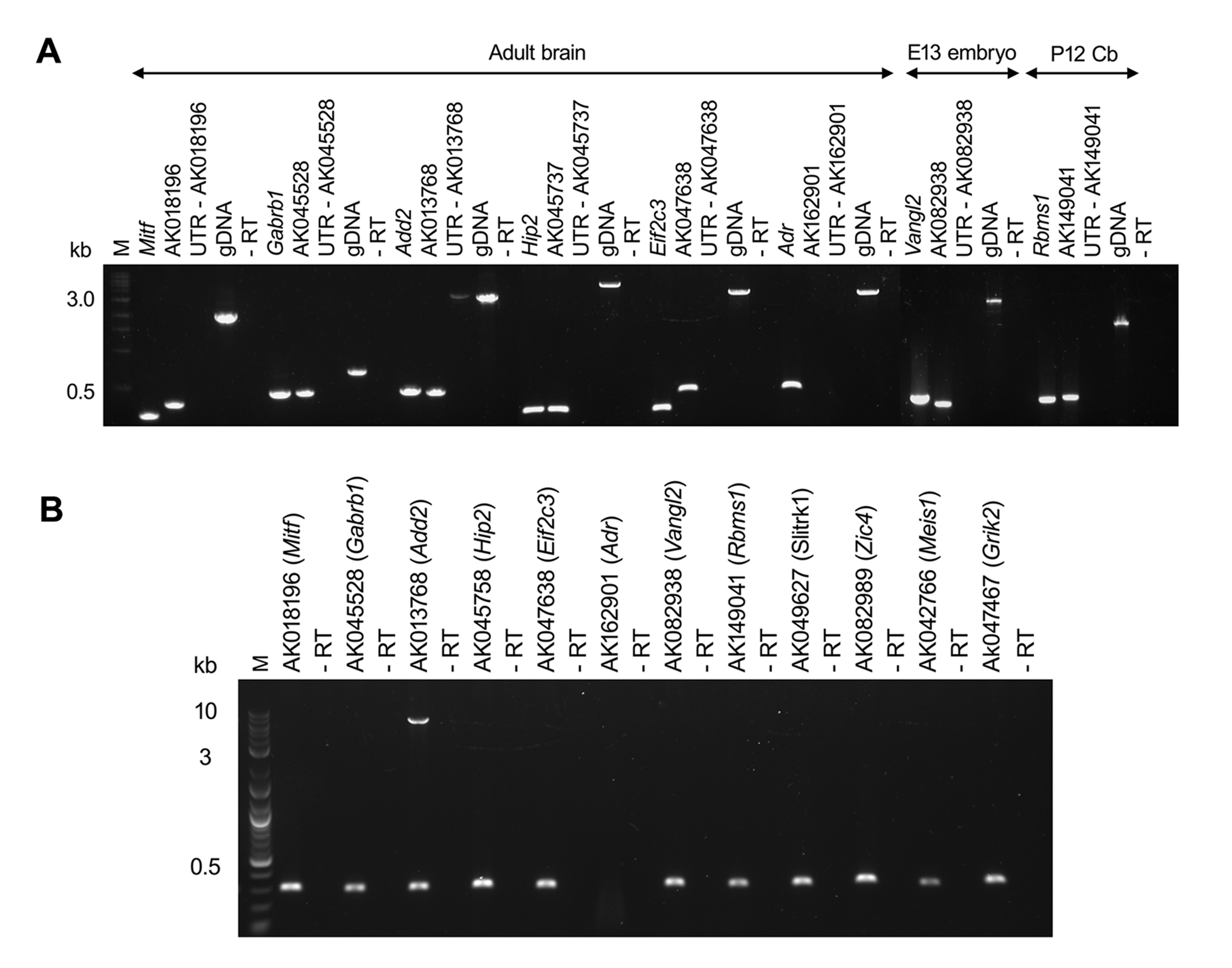

Supplement: Figure S4 — RT-PCR and 5′ RACE analysis of protein-coding and non-coding transcripts. (A) Total RNA was purified from the tissues and the developmental time-points indicated. RT-PCR was performed using primers spanning from the 3′ UTR of the protein-coding gene to the adjacent ncRNA genomic sequence. Control amplification using the same primer pairs from genomic DNA (gDNA) and a reaction containing no reverse transcriptase (-RT) is also shown. Importantly, RT-PCR of each protein-coding gene and ncRNA was performed from the same tissue. Apart from Add2/AK013768, no evidence for read-through from the 3′ UTR to the ncRNA was observed that would account for the in situ hybridisation results obtained (Figure 5, Figure 6). (B) 5′ RACE products of all 12 ncRNAs analysed in this study (adjacent pc genes are indicated in brackets). Total RNA was purified from the tissue corresponding to the in situ hybridisation data: adult brain (AK018196 - AK162901), P12 cerebellum (AK149041, AK042766 and AK047467) and E13.5 brain (AK082938, AK049627 and AK082969). In these reactions, a nested reverse primer approximately 300 bp from the predicted ncRNA transcription start site and a nested forward primer specific for the cap-ligated RACE anchor primer was used. A reaction containing no reverse transcriptase (-RT) is also shown for each primer pair. RACE reactions containing no TAP enzyme showed no amplification products (data not shown). (1.16 MB TIF) [file pgen.1000617.s004.tif]
