# Supplementary material for: Genomic and Transcriptional Co-Localization of Protein-Coding and Long Non-Coding RNA Pairs in the Developing Brain
Source: PLoS Genet. 2009 Aug 21;5(8):e1000617. doi: 10.1371/journal.pgen.1000617 (PMC2722021; doi:10.1371/journal.pgen.1000617)
Supplement: Figure S2 — Dinucleotide distribution analysis of CAGE tag starting sites with varying amounts of CAGE tag support for long ncRNAs (panels A and B) and their adjacent protein-coding transcripts (panels C and D), partitioned according to whether the long ncRNA is expressed in the brain (panels A and C) or elsewhere (panels B and D). Shown are the different [−1, +1] dinucleotides relative to each CAGE tag starting sites in the data set (note that the −1 nucleotide is not part of the sequenced tag). These cases were subdivided according to the numbers of tags supporting the CAGE tag starting sites (1,2,3 to 9 tags, and >9 tags). (0.03 MB DOCX) [file pgen.1000617.s002.doc]

**B**

**A**

**C**

**D**
