# Supplementary material for: Genomic and Transcriptional Co-Localization of Protein-Coding and Long Non-Coding RNA Pairs in the Developing Brain
Source: PLoS Genet. 2009 Aug 21;5(8):e1000617. doi: 10.1371/journal.pgen.1000617 (PMC2722021; doi:10.1371/journal.pgen.1000617)
Supplement: Text S1 — Functional associations and transcript read-through. (0.03 MB DOC) [file pgen.1000617.s011.doc]

**SUPPLEMENTAL TEXT**

**Functional associations and transcript read-through**

Here we consider the issue that even though we could experimentally exclude read-through from a majority of a random subset of transcripts, the minority that is due to read-through may be sufficiently numerous to give rise to significant functional biases. If so, these would be artifactual rather than pointing to functional biases in independently-transcribed non-coding RNAs.

In the main text we show that 11 out of 12 randomly picked non-coding transcripts (nctx) are transcribed independently from their neighboring protein-coding (pc) gene, and are not the product of protein-coding transcript read-through (rt). (Here we use the term *non-coding transcript* in an operational sense, i.e. those transcripts that were classified as independently transcribed non-coding transcripts by our filters, and thus include un-recognized splice variants of pc genes, and read-through from such genes.) We also show that the genomic distribution of nctx is biased in favor of them being transcribed in the genic vicinity of pc genes with particular functional annotations, the numerically most striking of which were *transcriptional regulation* and *nervous system development,* each showing over 3-fold enrichment.

The protein-coding genes with these functional annotations are highly expressed in brain tissue, e.g. 4.4% of all pc-transcripts reported in Novartis' GNF expression data set for "whole brain" tissue carry a *transcriptional regulation* annotation. Therefore, the fraction of rt-generated nctx that abut these protein-coding genes may be expected to show a similar over-representation in brain tissue, within the expected ~10% of all rt-generated nctx. This over-representation will be modest (in fact, less than ~10%) when expressed as a fraction of all nctx in brain tissue. However, this modest over-representation might still give rise to a substantial *fractional* over-representation of nctx abutting protein-coding genes with e.g. transcriptional regulation annotations. This will be the case, for example, if the expected fraction of such brain-associated nctx is low, as will be the case for annotations supported by few protein-coding genes.

As a null model, therefore, we postulate that (1) a fraction *f* of N nctx (operationally defined) instead derive from rt; (2) that the remainder N(1-*f*) are transcribed independently from loci distributed randomly across the genome; and (3) that the contribution due to rt from a particular pc gene is proportional to that gene’s level of expression in the tissue under consideration.

Assumption (1) quantifies the extent of rt, which we expect to be close to 10%; (2) specifies the core assumption we shall want to reject, namely that independently transcribed nctx have no association with their neighboring pc gene. Assumption (3) is arguably the simplest way to postulate a quantitative link between tissue-specific expression levels and the extent of rt. It is important to note that if transcriptional read-through is to be explanatory of our findings, we must have 0 < *f* < 1.

Let A denote an annotation, represented by a collection of pc genes; let g(A) denote the fraction of the genome covered by the genic environments of the genes in A (the "genic territory"); and let e(A) be the total expression level of transcripts of these genes, relative to the grand total of pc-expression in the tissue under consideration. We used the un-normalized (linear-scale) intensity readings from Novartis' GNF gene atlas to calculate e(A). For the genic territory in the definition of g(A) we used all sites that were (i) intergenic, and (ii) for which gene A was the nearest protein-coding gene.

By assumption, and without rt, the expected number of nctx within A’s genic territory is N g(A) under the null. With rt, this number is N (1-f) g(A) + N f e(A), since by assumption a fraction f of nctx will follow the expression patterns of pc genes. If, relative to the expectation g(A), an over-representation by a factor α is observed, we obtain the equation

N (1 – *f* ) g(A) + N *f* e(A) = α N g(A)

or

*f* = ( α – 1 ) ( e(A) / g(A) – 1 )^-1^.

Because the nctx in our set have a tight length distribution, we are justified in approximating the relative *number* of nctx by the proportional *coverage* in bases, which allows us to use for α the relative over-representation from our sampling procedure. To be conservative, we used the 95% confidence limits for the expected coverage as baselines, rather than just the expectation.

For A=*transcriptional regulation* (3955 locuslink identifiers; GO term 0045449), g(A)=0.03566, e(A)=0.0438 (1853247.3 / 42349351.75; GNF tissue “whole brain”), α=3.52 (1.90-9.17) This gives *f* = 11.0 (3.94 - 44.0).

For A=*nervous system development* (242 locuslink identifiers; GO term 0007399), g(A)=0.01844, e(A) = 0.00498 (210834.2 / 42349351.75; GNF tissue “whole brain”), α=3.41 (1.52 - 17.3). This gives *f* = -3.3 (-22.3 - -0.71).

Clearly, as both values of *f* fall outside of the range 0 < *f* < 1, no proportion of rt-derived nctx will have an effect that is sufficiently strong to explain the functional associations that we observe. In the case of nervous system development, the relative expression of protein-coding genes with this annotation is in fact *lower* than the fraction of the genome covered by the relevant genes (0.00498 vs. 0.01844); which is not unreasonable as developmental genes are by annotation expected to be highly expressed in developing, not adult, brain tissue; therefore, by assumption, any contribution of rt would in fact *decrease* the relative representation of this class, which is the opposite of what is observed. **In conclusion, we can dismiss rt as the sole factor influencing the observed functional biases in nctx distribution.**
